# Physical interpretation of shear-rate behaviour of soils and geotechnical solution to the coefficient of start-up friction with low inertial number

**DOI:** 10.1038/s41598-020-69023-w

**Published:** 2020-07-22

**Authors:** Jianbo Fei, Yuxin Jie, Xiaohui Sun, Hao Xiong

**Affiliations:** 1 Underground Polis Academy/Key Laboratory of Coastal Urban Resilient Infrastructures (MOE), Shenzhen University, Shenzhen, 518060 People’s Republic of China; 2 State Key Laboratory of Hydroscience and Engineering, Tsinghua University, Beijing, 100084 People’s Republic of China

**Keywords:** Civil engineering, Applied physics, Particle physics

## Abstract

Shear experiments on soils have revealed the effects of shear rate, confining pressure, and grain size on the residual shear strength, but their nature is not well understood. To interpret these behaviours, a single dimensionless inertial number *I* from granular physics is introduced. A linear relationship between coefficient of residual friction *μ*_*r*_ and the natural logarithm of *I* was found by analysing geotechnical test data from other literature and helps to resolve the *μ*(*aI*)-rheology, which was proved invalid in the quasi-static regime. A method is proposed that introduces two three-dimensional yield criteria for soils to classify the frictional properties between grains in the quasi-static regime. The empirical coefficient of start-up friction is replaced by strength parameters of the soil. When compliant with the Mohr–Coulomb yield criterion, this coefficient is positively correlated with the internal angle of friction but negatively correlated with the Lode angle. Moreover from further analysis, the calculated strength is smallest in the pure tension state, largest in the pure compression state, and intermediate in the pure shearing state. This result is consistent with the properties of compressive endurable and tensive intolerable for natural geomaterials.

## Introduction

The residual shear strength of a geomaterial is the minimum strength required to resist an external force. It represents the strength of granules undergoing fast shearing in the geomechanically sense or slow shearing in the granular physical sense, because the same value of shear speed is regarded diversely as fast and slow in the respective study of geomechanics and granular physics. The dependence of the residual shear strength on the displacement rate as well as the confining stress has been reported in a number of geotechnical studies over the last few decades^1–12^. Understanding the effect of shear rate and confining stress on shear strength is important in understanding the initiation of landslides and provides furthermore a potential early warning criterion.

There have been strong debates regarding the influence of shear rate on residual strength, considering the inconsistent test results reported concerning different soil types and test conditions. Positive correlations between residual strength and shear rate for clayed soils were reported several decades ago^1–5^; for example, Suzuki and co-workers^5^ found kaolin to have shear rates of 0.02–2.00 mm/min. This result contrasts with the above findings, even though positive and neutral effects on the rate were found for all vertical stresses and displacement rates no greater than 100 mm/min with London clay by Carrubba and Colonna^4^. Negative effects on the rate were noticed with Cowden till at certain confining stresses and shear rates because of strain softening. In addition, three types of residual strength were observed (positive, neutral, and negative) in slow shear tests under draining conditions by Tika and co-workers^6^. Lupini and co-workers^7^ found three types of residual shear behaviours—sliding, transitional, and turbulent—and correlated the divergent shear rate on the coefficient of residual friction to a type of shear behaviour, specifically, an increasing coefficient of residual friction with shear rate in the sliding mode and a decreasing or constant coefficient in the turbulent mode. The negative or neutral correlation in the turbulent mode was likely because the pressure generated in pore water was also rate dependent in a complicated manner^8^, and the effective stress (indicating a direct interaction between grains) derives by deducing the pore water pressure from the total stress following the principle of effective stress^9^. The turbulent mode is physically complex and may not follow a prescribed law, and hence is not discussed in the present paper.

Apart from the shear rate effect, Tika^10^ and Tika and Hutchinson^11^ pointed out other factors that influenced the residual shear strength, such as the magnitude of the confining stress, physical properties of soils such as grain size, and the properties associated with friction between grains. Consistently, Tika and co-workers^6^ and Carrubba and Colonna^4^ reported that the residual shear strength depends highly on confining stress, and a negative correlation between residual shear strength and confining stress was found^12^.

The angle of friction reflects the combined action of friction between particles and the degree of interlocking^13^. At slow shear rates when rolling or a transition of the particles dominate shear movement, it is revealed that the coefficient of residual friction is equivalent to the angle of friction^14^. Chattopadhyay^15^ proposed a non–linear law for the angle of residual friction $\varphi_{r}$ and the effective confining stress $\sigma^{\prime}$,

1 $$\tan \varphi_{r} = \frac{{K\tau_{r} }}{{\sqrt[3]{{\sigma^{\prime}}}}}$$

where *K* denotes an empirical parameter, and $\tau_{r}$ the residual shear strength. We rearrange this expression and obtain for the coefficient of residual friction $\mu_{r}$,

2 $$\mu_{r} = \frac{{\tau_{r} }}{{\sigma^{\prime}}} = \frac{1}{K}\tan \varphi_{r} \cdot \left( {\sigma^{\prime}} \right)^{{ - \frac{2}{3}}}$$

This law indicates a linear correlation between $(\sigma^{\prime})^{{{ - }\frac{2}{3}}}$ and the coefficient of residual friction $\mu_{r}$. Another non–linear law correlating the residual shear strength with the effective confining stress was proposed by Mesri and Shahien^16^,

3 $$\mu_{r} = \tan \varphi^{\prime}_{r} \cdot \left( {\frac{{\sigma^{\prime}_{p} }}{{\sigma^{\prime}_{{}} }}} \right)^{k}$$

where $\sigma^{\prime}_{p}$ denotes the pre-consolidated pressure, $\varphi^{\prime}_{r}$ the secant angle of residual friction at $\sigma^{\prime}_{{}} = \sigma^{\prime}_{p}$, and *k* the exponent of the ratio $\sigma_{p}^{^{\prime}} /\sigma^{\prime}$ that is empirically determined, ranging in value from 0 to 1.

Despite decades of research in the field of geotechnics engineering confirming the relevance of friction on the shear rate, the confining pressure, and grain diameter, the physical nature associated with the effect of shear rate and confining pressure on the coefficient of residual friction is not well understood. The present paper introduces the concept of the inertial number, which was initially developed in the field of granular physics, and tries to interpret the effects of shear rate, confining pressure, and grain size on soil strength from a physical viewpoint. This latter perspective could be more physically realistic than the geotechnical viewpoint. Conversely, the law obtained by inputting the geotechnical testing data of soils may serve as a solution to fixing invalid assertions from the *μ*(*I*)-rheology pertaining to the quasi-static regime. Hence, a possible regularized form of this *μ*(*I*)-rheology may be achievable. In addition, the present paper also proposes a method to evaluate the coefficient of start-up friction with the introduction of the three-dimensional yield criterion for natural soil. The strenuous effort expended when calibrating the empirical coefficient of friction is resolved using the strength parameter as a more convenient substitute. A brief introduction to the inertial number is presented in the next section.

## Inertial number from granular physics

According to da Cruz and co-workers^17^, a sheared granular system is closely related to the ratio of the shear rate to stress, and the coefficient of friction *μ* is controlled by a single non–dimensional parameter, namely, the inertial number *I*,

4 $$I = \frac{{\left| {\dot{\gamma }} \right|d}}{{\sqrt {P/\rho_{s} } }},$$

where *d* denotes the particle diameter, $\rho_{s}$ the particle density (or solid density), *P* the confining pressure, and $\dot{\gamma }$ the strain rate. The Savage/Coulomb number equals the square of the inertial number and was initially developed to describe the ratio of collisional stress to total stress for rapid granular flows^18,19^. The inertial number is basically a ratio of two time scales, one microscopic and the other macroscopic. The microscopic time scale $t_{micro}^{{}}$ refers to the duration of a grain of density $\rho_{s}$ in transiting a hole of size *d* under a confining pressure *P*, i.e., a microscopic rearrangement scale. To establish the relevance of this physical concept, we start with Newton’s law of motion,

5 $$m\frac{{d^{2} z}}{{dt^{2} }} = F_{z}$$

As $m \sim \rho_{s} d^{3}$, $\frac{{d^{2} z}}{{dt^{2} }} \sim \frac{d}{{t_{micro}^{2} }}$, and $F_{z} \sim Pd^{2}$, a substitution into the above yields

6 $$t_{micro}^{{}} = d/\sqrt {P/\rho_{s} }$$

The relative speed of the upper layer of grains compared with that of the lower layer is $\Delta u = \dot{\gamma }d$, then the mean time duration for a grain to move from one hole to another is

7 $$t_{macro} = {d \mathord{\left/ {\vphantom {d {\dot{\gamma }d}}} \right. \kern-\nulldelimiterspace} {\dot{\gamma }d}} = \dot{\gamma }^{ - 1}$$

Therefore, the inertial number expressed mathematically becomes

8 $$I = \frac{{t_{micro} }}{{t_{macro} }} = \frac{{\left| {\dot{\gamma }} \right|d}}{{\sqrt {P/\rho_{s} } }}$$

A small inertial number (i.e., $I \to 0$) implies that the macroscopic deformation is much smaller than the microscopic rearrangement, i.e., the quasi-static regime for the motion of a particle in physics, and is associated with the start-up stage of slope failures in geophysics. Note that during the collapse process of most slope failures, the inertial number become larger as the shear rate increases.

In the dense flow regime for granules sliding down inclined planes, the mathematical relationship between the coefficient of friction and *I* in the neutral range has been checked using numerical simulation methods such as the discrete element method and laboratory experiments, and a phenomenological analytical expression (i.e., *μ*(*I*)-rheology) has been proposed^20,21^,

9 $$\mu (I) = \mu_{s} + \frac{{\mu_{2} - \mu_{s} }}{{I_{0} /I + 1}}$$

where $\mu_{s}$ and $\mu_{2}$ denote the start-up (or lower) and upper limit values for the coefficient of friction, and *I*_0_ an experimental constant. Jop and co-workers^22^ extended the *μ*(*I*)-rheology to a full tensor constitutive relationship that links the strain rate to the deviatoric stress. Integrated with the governing equations of a continuum, the well-developed *μ*(*I*)-rheology has proven to be effective and powerful in the simulation of different types of granular motions, such as slumping^23^ and confined flows^24^.

Despite the success, limitations of the rheology emerge in the quasi-static regime when the inertial number approaches zero. The finite thickness of the confined flows and the exponential tail of heap flows in the experiment cannot be captured using the *μ*(*I*)-rheology when *I* approaches 0^25^. In addition, Barker and co-workers^26^ found that the governing equations are ill-posed if the inertial number is too small or too large. These invalidations indicate that the simple *μ*(*I*)-rheology is not physically realistic in the quasi-static regime, and an additional physical regularized form of the *μ*(*I*)-rheology in the limit of low inertial number is essential.

We shall next use the geotechnical parameter *d*_50_ (i.e., the nominal grain size) to represent parameter *d* in Eq. (4), and integrate *d*_50_, $\dot{\gamma }$, *P*, and $\rho_{s}$ to form a single physical parameter that can be used to classify natural soils,

10 $$I = \frac{{\left| {\dot{\gamma }} \right|d_{50} }}{{\sqrt {P/\rho_{s} } }}$$

In geotechnical sieve test, percentage retained on each sieve *p* is calculated as the ratio of the weight of soil retained to the total soil weight. Thus, the percentage finer than sieve size *PF* can be obtained by subtracting the cumulative percent retained ${\Sigma }$*p* in each sieve from 100% as

11 $$PF = 100\% - \sum p$$

In practice, the sieve analysis for soil is conducted by drawing a cumulative grain size distribution graph (curve), in which the ordinate represents the cumulative percent passing that equals to *PF* and the abscissa represents the particle size. In the graph, the nominal grain size *d*_50_ corresponds to the cumulative percent passing of 50%, which indicates half the grains are larger and the rest are smaller than the size.

Hence, with the input of the test data from the geotechnical apparatus, the relevance of the physically reasonable parameter (i.e., inertial number *I*) and the strength parameter can be explored, and the correlation between *I* and the coefficient of residual friction *μ*_*r*_ can be quantitatively determined.

## Correlation between *I *and *μ*_*r*_ from test data

**Figure 1:**
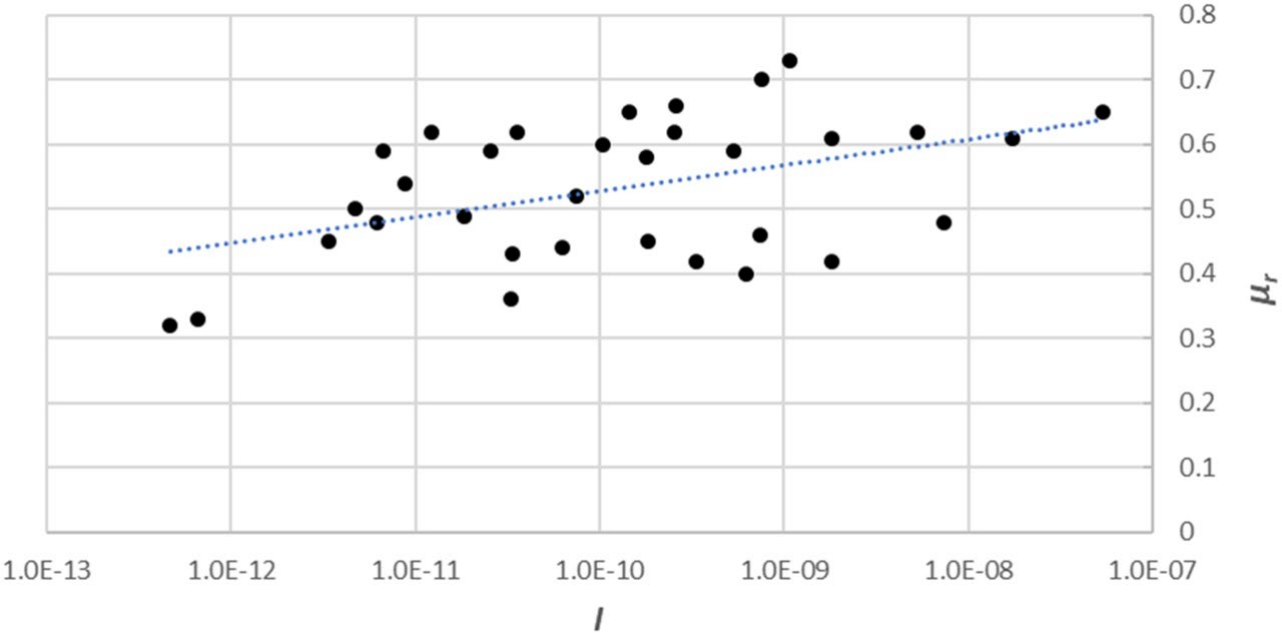
Relationship between inertial number *I* and coefficient of residual friction *μ*_*r*_ using the data obtained by Li and co-workers^28^.

In most cases, the unstable slope deforms initially at extremely small rates and may then accelerate to form a rapid-flowing avalanche at extremely large speeds. The displacement rates of the deforming slopes at different sliding states vary by an order of magnitude^27^. Li and co-workers^28^ conducted direct shear tests at shear rates with different orders of magnitude (i.e., 0.06, 0.6, and 6.0 mm/s) under different confining stresses (i.e., 50, 100, and 200 kPa) on mixtures of kaolin (grain size *d* < 2 × 10^−6^ m) and glass beads (grain size ranged from 6.3 × 10^−5^ m to 8 × 10^−3^ m). Different sets of specimens were set up by blending kaolin and glass beads in various proportions (100%–0%; 80%–20%; 60%–40%; 50%–50%; 40%–60%; 20%–80%; 0%–100%). With reference to the test results, the coefficient of residual friction was found to decrease with increasing confining stress but increased with shear rate and grain diameter. To explore the physical essence of the experimental results, we fitted the data of the inertial number *I* and coefficient of residual friction *μ*_*r*_ (Fig. 1), to visualize their correlation.

**Figure 2:**
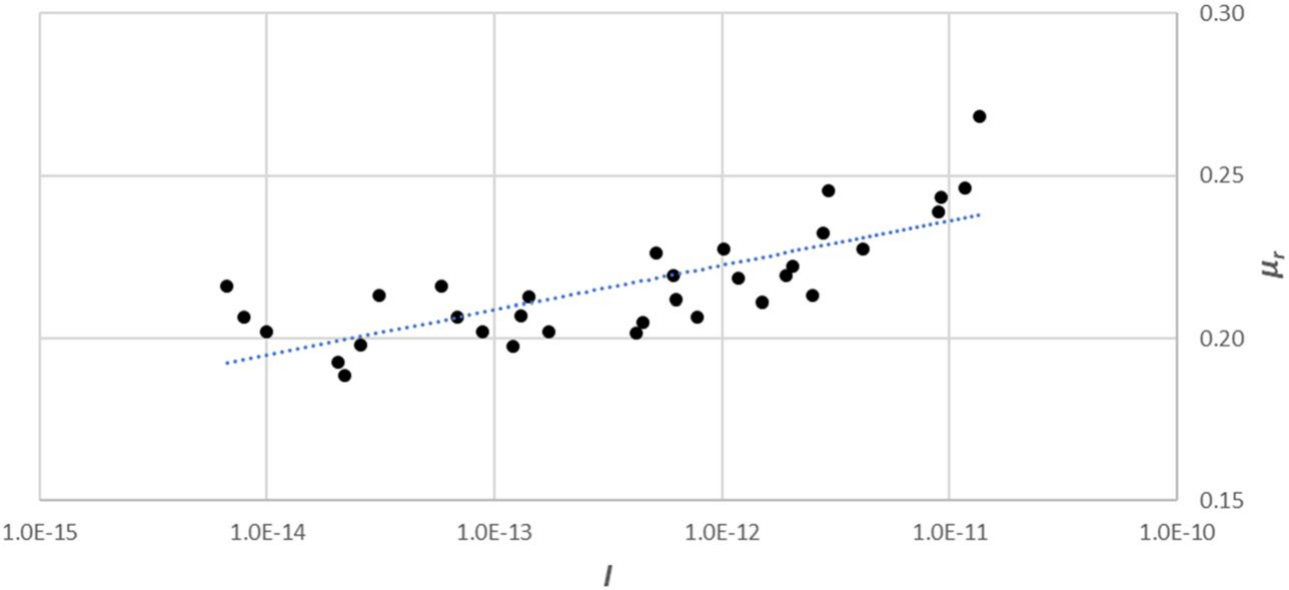
Dependence of the coefficient of residual friction on the inertial number compared with the shear rate obtained by Scaringi and Di Maio^29^.

**Figure 3:**
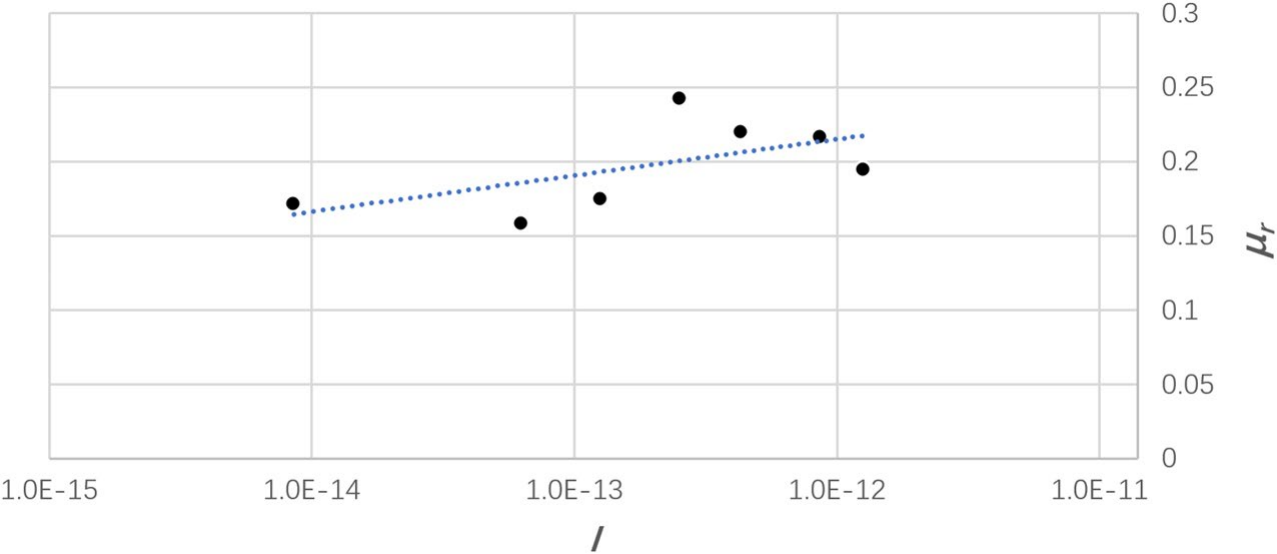
Data from Suzuki and co-workers^5^ illustrating the dependence of the coefficient of residual friction *μ*_*r*_ at low shear rate to the inertial number *I*.

Scaringi and Di Maio^29^ performed laboratory tests on kaolin (*d*_*50*_ ≈ 0.7 × 10^−6^ m; 74% clay, 25% silt, and 1% sand) using different test devices, in which the specimens were first subjected to given normal stresses (i.e., 125, 150, 205, 285, 305, and 350 kPa) and shear rates of different magnitudes ranging from 0.00011–66.5 mm/min. Note that according to AASHTO (ASTM D3282-09)^30^, the grain size of sands is the between 2 × 10^−3^ to 7.5 × 10^−5^ m, that of silts is 7.5 × 10^−5^ to 2 × 10^−6^ mm, and that of clays is smaller than 2 × 10^−6^ mm. The correlation between *I* and *μ*_*r*_ is displayed in Fig. 2. Suzuki and co-workers^5^ conducted ring shear tests on kaolin (*d*_*50*_ = 7 × 10^−6^ m, maximum diameter *d*_*max*_ = 2 × 10^−4^ m) and mudstone (*d*_*50*_ = 2 × 10^−5^ m, *d*_*max*_ = 8.5 × 10^−4^ m) varying the shear rate over different orders of magnitude in the range 0.02–2.0 mm/min; the confining pressure was fixed at 196 kPa. The relationship between *I* and *μ*_*r*_ was also plotted by inputting test data (Fig. 3).

We see from Figs. 1, 2 and 3 that, despite the scatter of the data points, *μ*_*r*_ roughly increases linearly with the natural logarithm of *I*, i.e.,

12 $$\mu_{r} = a + b\ln (I)$$

where the fitting parameters are *a* = 0.9299 and *b* = 0.0175 from Li and co-workers^28^, *a* = 0.5085 and *b* = 0.0106 from Suzuki and co-workers^5^, *a* = 0.3867 and *b* = 0.0059 from Scaringi and Di Maio^29^.

**Figure 4:**
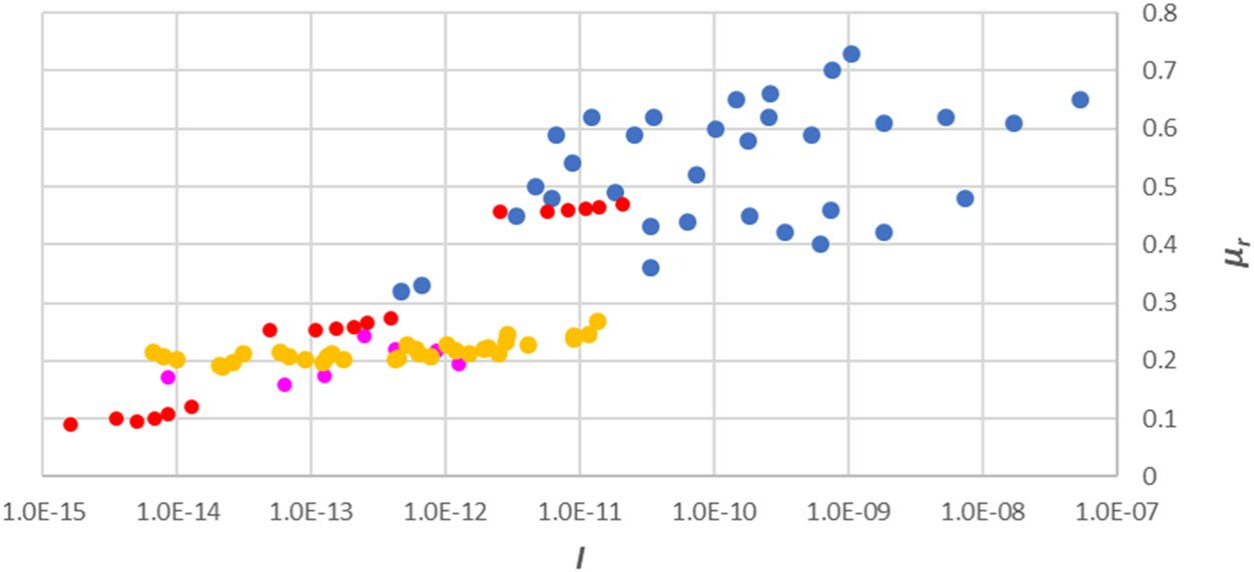
Normal-log plot of the coefficient of residual friction at low shear rate and the inertial number using data obtained by: Bhat and Yatabe^31^: red; Suzuki and co-workers^5^: pink; Scaringi and Di Maio^29^: yellow; and Li and co-workers^28^: blue.

Three soil samples taken from distinctive natural landslide areas by Bhat and Yatabe^31^ were composed of clay, silt and sand, whose content weight percentage were 21%–60%–19%, 20%–68%–12%, and 24%–55%–21%, respectively. By including the data set from Bhat and Yatabe^31^ with the above mentioned three sets of data in a single graph (Fig. 4), we obtain the entire correlation between *I* and *μ*_*r*_ at different shear rates obtained from a variety of experiments on diverse soil types using different equipment. As a whole, these experimental data also generally indicate a linear relationship between the natural logarithm of *I* and *μ*_*r*_.

It deserves to compare our results with the well-posed regularized form of the *μ*(*I*)*-*rheology below the low inertial number limit proposed by Baker and Gray^32^. They used the natural logarithm function based on their neutral stability analysis to describe the power-law decay of *μ* as *I* approaches 0, expressed in the form

13 $$\mu (I) = \alpha \left[ {\ln (A \cdot I^{ - 1} )} \right]^{ - 0.5}$$

where *α* and *A* denote two fitting parameters. Different from our finding, Eq. (13) describes $e^{{ - \alpha^{2} /\mu^{2} }}$ is proportional to *I*. It is also noted that Eq. (13) diverges when *I* approaches 1.

It also deserves mentioning that the fitting parameters *a* and *b* are obtained by inputting the geotechnical testing data with various kinds of soils from other literatures. These two empirical parameters change with the type of soils and can serve as indicators for the material properties. Considering the complexity of natural soils, we admit that the acquirement of a universal fitting solution with constant fitting parameters that is applicable to different types of soils is still challenging.

## Adoption of three-dimensional yield criterion for soil

With the focus on granular motion in the quasi-static regime, the previous two sections investigated the frictional behaviours of natural soils with extremely small inertial number. As inertial numbers corresponding to the quasi-static regime is negligibly small compared with that in the rapid flow regime, a common way of covering the complex frictional behaviour when modelling rapid granular flow is the use of a simple parameter, i.e., the coefficient of start-up friction $\mu_{s}$. In contrast to the above sections, this section seeks the description of the physical parameter $\mu_{s}$ from a geotechnical perspective.

Natural avalanches are made of geomaterials with complex compositions. Considering the test materials of the *μ*(*I*)-rheology are initially homogeneous dry grains (e.g., sands and glass beads), the parameter settings may be ineffective for natural avalanches that are composed of complex sediments with branching grain sizes. In addition, the three empirical parameters in the *μ*(*I*)-rheology, i.e., *μ*_*s*_, *μ*_*2*_, *I*_*0*_, were obtained in the calibration of a large number of test data, which is time-consuming and inconvenient. This section suggests a simple and practical method to obtain the coefficient of start-up friction for natural geomaterials, inspired by the traditional non-Newtonian fluid, the Bingham fluid, and the Savage–Hutter (S–H) model^33^, for which the yield criterion is reached when a mass is on the move. Two widely recognized three-dimensional yield criteria for geomaterials—the Drucker–Prager criterion and the Mohr–Coulomb criterion—are introduced to describe the coefficient of friction in the quasi-static regime. At the same time, the deviatoric stresses still obey a constitutive framework proposed by Jop and co-workers^27^, i.e., they are related to the strain rate, confining pressure, and coefficient of friction. There, the strength parameter values of natural materials, which are readily available, replace the empirical coefficient of start-up friction derived from experiments.

### Drucker–Prager criterion

The Drucker–Prager criterion^34^ can reveal the influence of the mean principle stress on the strength of a geomaterial. The three-dimensional yield criterion can be mathematically expressed as

14 $$\sqrt {J_{2} } - \alpha_{m} I_{1} - K_{m} = 0$$

where *a*_*m*_ and *k *_*m*_ denote the strength parameters. *k*_*m*_ is a material constant related to cohesive strength, for cohesionless granular material *k*_*m*_ = 0. Following Jop and co-workers^27^, we suppose that the internal stress tensor is linearly dependent on the strain rate tensor^35,36^, then the internal stress tensor of a moving granular mass is expressed as

15 $$\sigma_{ij} = - P\delta_{ij} + \tau_{ij}$$

16 $$\tau_{ij} = \frac{{\mu_{s} P}}{{\left| {\dot{\gamma }} \right|}}\dot{\gamma }_{ij}$$

where $\dot{\gamma }_{ij}$ is the strain rate tensor, and $\left| {\dot{\gamma }} \right| = (\dot{\gamma }_{ij} \dot{\gamma }_{ij} /2)^{1/2}$ is the second invariant of $\dot{\gamma }_{ij}$. Equation (16) describes that the shear stress depends not only on the product of pressure and coefficient of friction (as in Coulomb-type frictional materials), but also on the shear rate (as the visco-plastic law). We then determine the first invariant of the normal stress *I*_*1*_, and the second invariant of the shear stresses *J*_*2*_ to be

17 $$I_{1} = \sigma_{1} + \sigma_{2} + \sigma_{3} = \sigma_{xx} + \sigma_{yy} + \sigma_{zz} = 3P$$

18 $$J_{2} = \frac{1}{2}s_{ij} s_{ji} = \frac{{3\mu_{s}^{2} P^{2} }}{{\left| {\dot{\gamma }} \right|^{2} }}(\dot{\gamma }^{2}_{xy} + \dot{\gamma }^{2}_{yz} + \dot{\gamma }^{2}_{zx} ) = 3\mu_{s}^{2} P^{2}$$

where *s*_*ij*_ is the deviatoric stress tensor. By substituting Eqs. (17) and (18) into Eq. (14), we find the coefficient of start-up friction is only related to the strength parameter *a*_*m*_,

19 $$\mu_{s} = \sqrt 3 \alpha_{m}$$

### Mohr–Coulomb criterion

The Mohr–Coulomb yield criterion is more accurate than the Drucker–Prager criterion for friction-dominated geomaterials and hence has been widely adopted in modelling and calculations of strength-related geotechnical problems. By including the two-dimensional Mohr–Coulomb yield criterion into the depth-averaged continuum governing equation, the S–H model has simulated successfully the rapid motion of shallow granular flow. The present paper introduces this same yield criterion, which we express as

20 $$\frac{{I_{1} }}{3}\sin \varphi - \sqrt {J_{2} } \left( {\frac{1}{\sqrt 3 }\sin \theta_{L} \sin \varphi + \cos \theta_{L} } \right) + c\cos \varphi = 0$$

in which *φ* denotes the angle of internal friction, *c* cohesion, and *θ*_*L*_ the Lode angle, which is determined from the ratio between the intermediate principal stress and the minor/major principal stresses. As the classical *μ*(*I*)-rheology is initially developed for cohesionless granular materials, the present paper neglects cohesion *c* in the calculation. Introducing Eqs. (17) and (18) into Eq. (20) produces

21 $$\mu_{s} = \frac{\sin \varphi }{{\sin \theta_{L} \sin \varphi + \sqrt 3 \cos \theta_{L} }}$$

**Figure 5:**
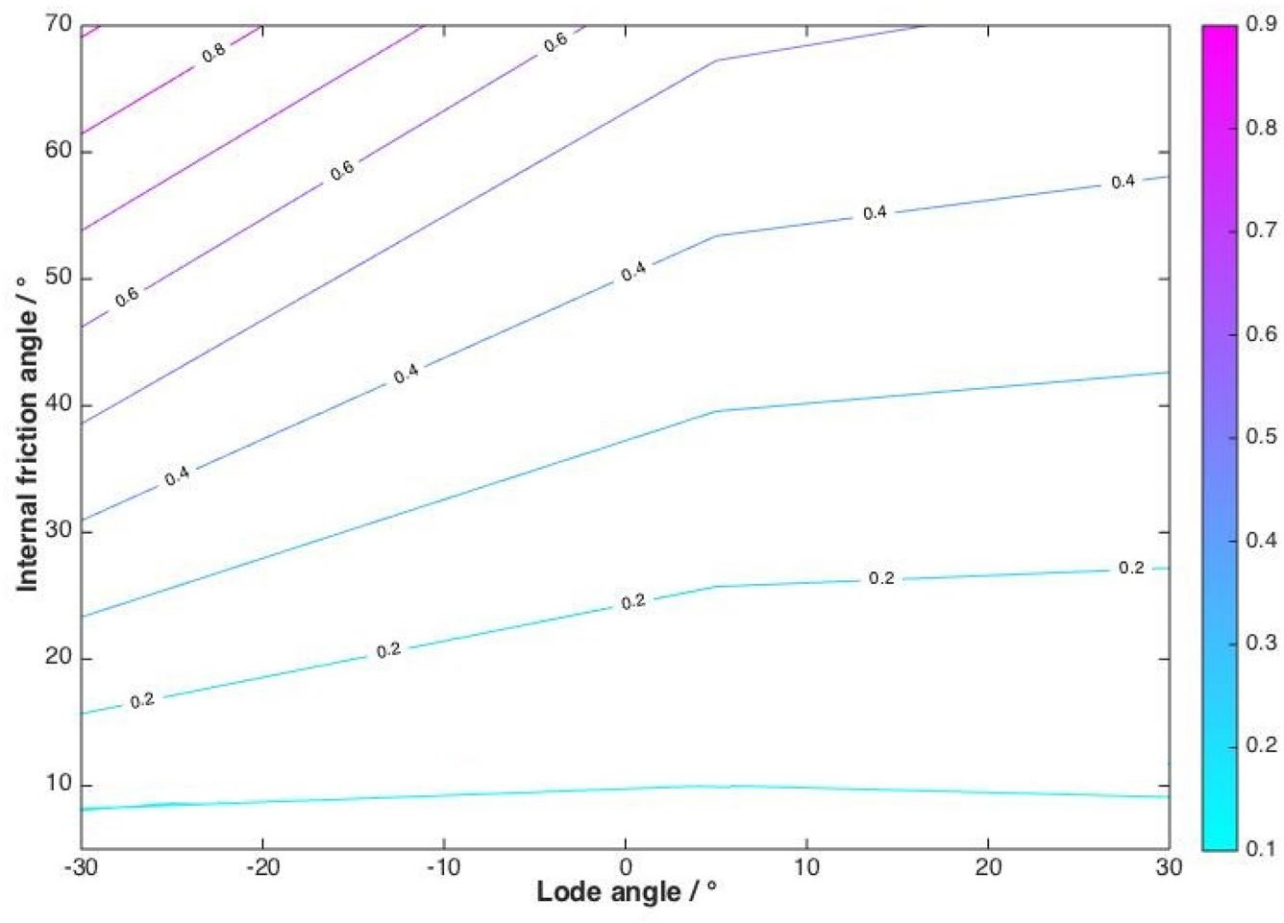
Contour lines of the coefficient of start-up friction as a function of internal angle of friction and Lode angle.

The neglect of cohesion limits the application of the derived equation to merely cohesionless granular materials. Based on Eq. (21), Fig. 5 displays the influence of the internal angle of friction and the Lode angle on the coefficient of start-up friction. It is noted that the empirical value of the coefficient of start-up friction *μ*_*s*_ obtained by Andreotti and co-workers^25^ ranged from 0.26 to 0.384, that by Barker and co-workers^26^ was 0.383, that by Pouliquen^37^ ranged from 0.378 to 0.422. Considering that the input range of the internal angle of friction (i.e., 5°–70°) covers the properties of most natural granular materials, the calculated range of the coefficient of start-up friction covers the empirical value obtained from the laboratory experiments^25,26,37^.

Moreover, in Fig. 5, the coefficient of start-up friction, which represents shear strength, increases with internal angle of friction but decreases with Lode angle. The Lode angles *θ*_*L*_ = – 30°, 0°, and 30° represent pure tension, pure shearing, and pure compression, respectively. Therefore, considering Eqs. (15) and (16), under the same confining pressure and with the same strain rate, we conclude that, to reach the yield state for a moving mass, small stresses are needed in the pure tension state, large stresses in the pure compression state, and median stresses in the pure shearing state. This is consistent with the real nature of the strength of granular materials such as soil, which well-resist compression but collapse easily when under tension.

### Conclusion

Understanding the nature of the start-up of landslides (or avalanches) has been a common focus in the communities of engineering geology, geotechnical engineering, and other related research areas. At present, the research in the field of "engineering geology" mainly starts from a geological site investigation, and focuses for example on the examination of geological properties of landslides and the analysis of hazard causes. In geotechnical engineering, research methods such as solid mechanics are commonly used to study the stress–strain properties assuming an "infinitesimal deformation" before the landslide occurs. Leaving aside the engineering viewpoint, the present paper introduced a widely accepted concept of “inertial number” from particle physics, and used it to interpret the effects of shear rate, confining pressure, and particle size on soil strength, thereby bridging the gap between engineering and physics. By introducing the single dimensionless number *I*, we explained a well-known phenomenon, that being the shear rate, confining stress, and grain size were fundamental influencing factors on the coefficient of residual friction. By analysing the test data obtained from laboratory experiments performed by Bhat and Yatabe^30^, Suzuki and co-workers^5^, Scaringi and Di Maio^29^, Li and co-workers^28^, a linear correlation was found between the natural logarithm of *I* and the residual shear strength. Considering the *μ*(*I*)-rheology with low inertial number is not physically realistic, this also helps to interpret the frictional properties of granular motion in the quasi-static regime.

Additionally, the present paper also provided a solution to determine the coefficient of start-up friction by introducing the three-dimensional yield criterion for soil. The main advantage is that the empirical law for the coefficient of friction, which required enormous effort and was hard to obtain for natural geomaterial, can be replaced by strength parameters for soils. Two expressions for the coefficient of start-up friction were derived using the Drucker–Prager and the Mohr–Coulomb yield criteria. The coefficient of start-up friction was found to be closely related to the strength parameter *a*_*m*_ when the Drucker–Prager criterion was introduced, and to the internal angle of friction along with the Lode angle when the Mohr–Coulomb yield criterion was adopted. When described by the latter, the coefficient of start-up friction is positively related to the internal angle of friction, but is negatively correlated with the Lode angle. The required yielding stress, that is, the smallest calculated in the pure tension state, the largest calculated in the pure compression state, and the median calculated in the pure shearing state, were consistent with the natural properties of the soil; i.e., geomaterials are good at resisting compression, rather than tension. Apart from the above findings, the following issues related to the physical interpretation of shear-rate behaviour of soils should also be noted:

1. The nominal grain size *d*_50_ was used in the present paper to represent the mean diameter of grains, and the test materials of clays were composed of grains for which the sizes cross several orders of magnitude. Nevertheless, the real nature of the complex composites may be more complicated than described by a law with the mean grain diameter parameter.
2. It can be concluded from Eq. (6) that the microscopic time scale regarding to the typical time scale of rearrangements is closely related to the grain size. Thus, we can see from Eq. (8) that the scale of inertial number varies consistently with that of grain size, which may explain the origins of complex phenomena such as size segregation. In the simulation using discrete element method (DEM), Rognon and co-workers ^38^ and Tripathi and Khakhar^39^ used a concept of local mean particle diameter *d* (related to the local particle volume fractions) to define *I* when describing a polydisperse granular media. To convey the macroscopic constitutive law of a polydisperse granular media, the present paper adopts the geotechnical concept of the nominal grain size *d*_50_ as an alternative to represent the equivalent mean diameter. Research results in this paper indicate that the rheology initially developed for grains of relatively uniform size could also be relevant to polydisperse granular media. Note that though the frictional properties of granular motion in the quasi-static regime is well interpreted by the introduction of “inertial number” given under the condition of relatively uniform soil grain size distribution, the effect of the uniformity of grain size on the inertial number as well as the *μ*(*I*)-rheology needs further investigation, considering multiscale grain sizes in natural soils.
3. Cohesion was neglected in the analysis of the relation between the residual shear strength and the inertial number because its magnitude is negligibly small compared with that of the confining pressure. Moreover, the derived expression for the coefficient of start-up friction is merely applicable to cohesionless granular materials such as sands. A more detailed analysis should take cohesion into consideration.
4. Negative or neutral correlation between the residual shear strength and shear rate cannot be reflected by the introduction of the inertial number because the physical law for the turbulent mode is complex and may not follow a perspicuous rule, most likely because we do not take into account the effect of water.
5. Broadly graded and irregular-shaped particles are often encountered in real granular materials used in industries and natural geomaterials in geophysics. The *μ*(*I*)-rheology was initially developed for a simple deformed granular system composed of nearly identical spherical grains that interact through solid contacts. The limitations cannot be denied when the rheology is applied to natural soils that are made of broadly graded and irregular shaped grains.The role of the particle shape has been considered in some studies (e.g., GDR MiDi^40^), and it is found that nonlocal effects become non-negligible owing to the irregular shape of particles. This has motivated the investigation of an effective nonlocal rheology, which is able to account for such geometric effects (e.g., Karmin and Koval^41^). In the discrete element method, one of the strategies to include the complexity of broadly gradation and irregular particle shape is gluing spherical particles together to create angular particles of various scales of size and shapes. Another strategy is including additional features to particle interactions such as the rotation effect to mimics the macroscopic behaviour of facetted grains^42^. In the introduced *μ*(*I*)-rheology that describes the constitutive law of a deforming granular continuum, the complex friction behaviors of broadly graded and angular soil particles can be characterized by different values or even mathematical expressions for the parameters *μ*_*s*_, *μ*_*2*_, and *I*_*0*_. So, the *μ*(*I*)-rheology initially found for spherical grains could reflect, to some extent, basic features of natural soils by adopting appropriate parameters.
